# Generalization of multisensory perceptual learning

**DOI:** 10.1038/srep23374

**Published:** 2016-03-22

**Authors:** Albert R. Powers III, Andrea Hillock-Dunn, Mark T. Wallace

**Affiliations:** 1 Kennedy Center, Vanderbilt University, Nashville, Tennessee, USA; 2 Department of Hearing and Speech Sciences, Vanderbilt University, Nashville, Tennessee, USA; 3 Neuroscience Graduate Program, Vanderbilt University, Nashville, Tennessee, USA; 4 Medical Scientist Training Program, Vanderbilt University School of Medicine, Nashville, Tennessee, USA; 5 Department of Psychiatry, Yale University, New Haven, Connecticut, USA

## Abstract

Life in a multisensory world requires the rapid and accurate integration of stimuli across the different senses. In this process, the temporal relationship between stimuli is critical in determining which stimuli share a common origin. Numerous studies have described a multisensory temporal binding window—the time window within which audiovisual stimuli are likely to be perceptually bound. In addition to characterizing this window’s size, recent work has shown it to be malleable, with the capacity for substantial narrowing following perceptual training. However, the generalization of these effects to other measures of perception is not known. This question was examined by characterizing the ability of training on a simultaneity judgment task to influence perception of the temporally-dependent sound-induced flash illusion (SIFI). Results do not demonstrate a change in performance on the SIFI itself following training. However, data do show an improved ability to discriminate rapidly-presented two-flash control conditions following training. Effects were specific to training and scaled with the degree of temporal window narrowing exhibited. Results do not support generalization of multisensory perceptual learning to other multisensory tasks. However, results do show that training results in improvements in visual temporal acuity, suggesting a generalization effect of multisensory training on unisensory abilities.

Our ability to perceive the world in an accurate and meaningful way depends critically upon the appropriate integration of stimuli from the different senses. One of the more challenging aspects of this process comes in determining which of a constant stream of stimuli from the different senses were caused by the same environmental event. One statistical feature that the brain employs in accomplishing this task is the temporal structure of the incoming sensory inputs. Thus, those events that occur in close temporal proximity are likely to have been caused by the same environmental event, whereas events that occur at disparate times are unlikely to have a common origin. However, because the environmental energies carrying information from the different senses (i.e., light, sound) propagate at different rates, the temporal relationship between these different inputs must be flexibly specified. For this reason the construct of a temporal window of multisensory binding has been proposed–an interval spanning several hundred milliseconds and within which paired events in two different sensory modalities are likely to produce enhanced neural, perceptual, and behavioral responses^1^,^2^,^3^,^4^,^5^,^6^,^7^,^8^,^9^.

Although these studies have characterized the multisensory temporal binding window in adults, and others have focused on its developmental dynamics^10^,^11^,^12^, only recently have efforts been made to examine the relative stability/lability of this window. This work illustrated marked malleability in multisensory temporal processes following perceptual training^13^, a plasticity that could be linked to activation changes in a multisensory cortical network centered on the posterior superior temporal sulcus^14^. Numerous questions remain about the perceptual changes induced by such training. Foremost among these are questions of generalization - specifically whether the improvements in multisensory temporal acuity brought about by training are capable of transferring to other tasks involving either multisensory or unisensory (i.e., visual alone, auditory alone) processing. Reports of such generalization are rare in the perceptual learning literature^15^,^16^,^17^,^18^,^19^,^20^, with relatively few studies showing effects that transfer to untrained tasks. Although one study has demonstrated improvements in auditory temporal processing following training on a somatosensory temporal discrimination task^20^, and some studies examining the effect of multisensory adaptation effects demonstrate some degree of cross-modal transfer^21^,^22^,^23^, few studies have shown transfer of multisensory perceptual learning^24^,^25^,^26^,^27^. One notable demonstration of these effects has described generalization of multisensory perceptual learning of a temporal order judgment (TOJ) task onto the sound-induced flash illusion (SIFI)^27^, although the degree to which these changes are driven by effects on unisensory or multisensory processing remain unclear.

To examine this question, we trained participants on two versions (i.e., a 2-alternative forced choice (2-AFC) and a 2-interval forced choice (2-IFC)) of an audiovisual simultaneity judgment task in which they received trial-by-trial feedback on their perceptual reports (published previously in ref.^13^; Fig. 1a,b). We then examined these participants’ performance on a different task known to be sensitive to the temporal structure of audiovisual stimuli –the sound-induced flash illusion (Fig. 1c)^28^,^29^.

## Results

### No change in SIFI illusion is seen after training, but improvements are seen in correctly recognizing two-flash conditions

Participants in both the 2-AFC and 2-IFC training and passive exposure groups completed the SIFI task immediately after the baseline simultaneity judgment assessment (i.e., prior to training) and again after the final simultaneity judgment assessment following the 5 days of training. The results of these experiments are summarized in Fig. 2. Here changes in the proportion of trials in which participants report two flashes are plotted as a function of the number of flashes and beeps actually presented during pre- and post-training assessments. Baseline between-group differences at each condition were not evident (F_5_ = 1.304, p = 0.26). Examination of training effects indicate significant main effects of group (F_2_ = 9.68; p < 0.001), training status (F_1_ = 13.01; p < 0.001), and condition (F_5_ = 34.05; p < 0.001) as well as significant group-by-training status (F_2_ = 9.68; p = 0.04), group-by-condition (F_10_ = 3.04; p < 0.001), and training status-by-condition (F_5_ = 13.81; p < 0.001) interactions. Post-hoc paired t-tests revealed significant increases in the likelihood of correctly identifying two-flash conditions in the 2-AFC (Fig. 2a; 2 Flash/0 Beep: t_21_ = 3.0, p = 0.037; 2 Flash/1 Beep: t_21_ = 2.86, p = 0.038; 2 Flash/2 Beep: t_21_ = 3.02; p = 0.033; all p values Holm-Sidak corrected for multiple comparisons) and 2-IFC groups (Fig. 2b; 2 Flash/0 Beep: t_19_ = 4.6, p = 0.001; 2 Flash/1 Beep: t_19_ = 3.75, p = 0.007; 2 Flash/2 Beep: t_19_ = 3.09; p = 0.02; all p values Holm-Sidak corrected for multiple comparisons). In contrast, there were no significant changes in likelihood of reporting two flashes when only one was present after training when plotted as a function of the number of beeps present across any conditions; this includes the 1 Flash/2 Beep (or SIFI “fission” illusion condition). Similarly, there was no change in the likelihood of reporting only one flash when two were present and paired with one beep, or the SIFI “fusion” illusion. The Exposure Group (Fig. 2c) did not exhibit any significant changes on any conditions after passive exposure to the stimulus pairings. Taken together, results indicate that while all groups show evidence of both the fission (1 Flash/2 Beep) and fusion (2 Flash/1 Beep) versions of the SIFI illusion as evidenced by comparison with other 1-Flash and 2-Flash conditions, respectively, neither of these illusory conditions were affected by training. In contrast, any increment in 2-Flash reporting exhibited by the training groups was shared among the 2-Flash conditions, supporting a specific effect on recognition of two flashes regardless of the presence, absence, or number of auditory stimuli.

### Performance improvements are driven by increased hits across all conditions

In each of the participant groups (2-AFC, 2-IFC, and passive exposure), there was no significant change in the proportion of false alarms following training (Fig. 3a). In contrast, significant increases were seen in the proportion of hits in the two training groups (2-AFC: t_21_ = 2.84, p = 0.0099; 2-IFC: t_19_ = 3.22, p = 0.0045) but not in the exposure group. Similarly, a significant increase in hits persisted in the 2-AFC participants who returned for follow-up assessment (Fig. 3b; t_13_ = 2.89, p = 0.0125). This group also demonstrated a decrease in false alarms on follow-up, manifested as a decrease in likelihood of reporting the SIFI (t_13_ = 2.49, p = 0.027).

### Multisensory perceptual training results in changes in sensitivity (d-prime) on the SIFI task, but no changes in response bias

In order to determine if the improvements in two-flash reporting seen with training were the result of a true sensitivity increase or due to a change in response bias, signal detection theory was employed. In this approach, measures of sensitivity (d-prime) can be derived from the correct and incorrect responses in the SIFI task. Individual baseline, post-training/exposure, and follow-up (as applicable) data are plotted in Fig. 4 for d-prime (a–c) as well as for measures of response bias (i.e., beta (d–f)) measures.

Analysis of baseline measures for each group indicated no group-wise difference in baseline d-prime (F_2,53_ = 1.27; p = 0.28) or beta (F_2,53_ = 0.21; p = 0.81). A main effect of training was detected on mixed ANOVA with training status (pre/post) as a within-subject factor and group as a between-subjects factor (F_1,2_ = 11.49, p < 0.001). In both the 2-AFC and 2-IFC training groups, marked increases in sensitivity were noted following training (2-AFC mean increase in d-prime = 0.45; t_21_ = 2.38, p = 0.027; 2-IFC mean increase in d-prime = 0.38; t_19_ = 2.34, p = 0.030) (Fig. 4a,b), and the 2-AFC group continued to demonstrate significant increases on follow-up assessment (main effect of assessment (follow-up cohort, n = 14), F_2_ = 11.49, p < 0.001; t_13_ = 4.33, p < 0.001). In contrast, the passive exposure group showed no difference in sensitivity at the two time points (Fig. 4c). In contrast to these changes, no significant changes in beta were noted among any of the three groups on initial or follow-up assessment (Fig. 4d–f).

Collectively, these results illustrate that while training on a multisensory simultaneity judgment task does not alter the perception of a temporally-dependent multisensory illusion (i.e., SIFI), it does result in an increased ability to correctly detect two flashes presented in close succession when compared with pre-training data. These results support a lack of generalization from the multisensory training for the illusory task, but do provide evidence for generalization from multisensory training for improvements in visual temporal perception.

### Increases in d-prime correlate with the degree of temporal window narrowing

To determine whether the degree of change induced in the multisensory temporal binding window brought about by training is able to predict the degree of change in sensitivity on the SIFI task, individual difference scores in multisensory temporal window size and d′ were entered as factors into a linear regression. As seen in Fig. 5a, there is a direct correlation between the decrease in window size and the increase in sensitivity immediately after training (r^2^ = 0.1967; p = 0.0145). In a related analysis, Fig. 5b highlights a significant correlation between participants’ pre-/post-training difference in probability of reporting audiovisual simultaneity on the AFC simultaneity judgment task at the 300ms SOA and the increase in d-prime observed at this SOA that was most significantly altered on the SIFI task (r^2^ = 0.2439; p = 0.0195).

## Discussion

In the current study we show that individuals who have undergone perceptual training on an audiovisual simultaneity judgment task do not demonstrate altered perception of the sound-induced flash illusion (SIFI) on those conditions in which the illusory percept is typically seen. Rather, they demonstrate performance improvements driven by an increase in the correct recognition of two-flash conditions after training. Moreover, we have demonstrated that the magnitude of change in performance on the SIFI task is directly dependent upon the degree of temporal window narrowing. Finally, we have shown that these changes are stable at least one week following the cessation of training.

The use of signal detection theory as an approach to the SIFI is unique, and here it is somewhat unconventionally applied. In contrast to traditional studies meant to determine individuals’ sensitivity to a faintly-presented signal, the SIFI task is meant to determine how susceptible individuals are to a stimulus that is in fact not present; thus, the effects of interest here (i.e., the illusion) would traditionally be described as false alarms (in the case of fission illusion) and misses (in the case of fusion illusion). Similar approaches have been taken to illusory phenomena^30^,^31^ (including the SIFI^27^) and hallucinations^32^, and applied here allow a more nuanced understanding of the interplay between unisensory and multisensory processing. In particular, the distinction between sensitivity improvements and response bias will be crucial in interpreting some of the differences observed here, as described below.

One of the more intriguing findings in the current study is the lack of a correlation between window narrowing and d-prime for participants trained on the 2-IFC task. One explanation for this difference is that because a single-presentation 2-AFC task of this type (as opposed to the 2-IFC construct) relies more upon the setting of an internal criterion^33^,^34^, this decrease may be the result of post-perceptual processes. Moreover, such a mechanism would likely result in a change in response bias (i.e., β) in the 2-AFC group, but no such change was found. A more plausible explanation for these differences may rest on subtle differences in attention inherent in the 2-AFC and 2-IFC tasks^35^,^36^,^37^. Because the 2-AFC task requires direct judgment of audiovisual simultaneity in a single stimulus pair rather than detection of timing differences between two objectively simultaneous and non-simultaneous pairs (as in the 2-IFC task), participants in the 2-AFC group may be more likely to attend to the fine temporal structure of single stimulus pairs, thus leading to both increases in hits and decreases in false alarms, a combination observed only in the 2-AFC group (although only on follow-up assessment). However, further studies with more sensitive measures of attention may be needed to determine whether these mechanisms may be at play in the differences observed here.

The observation that performance improvements after multisensory perceptual learning are seen not in another audiovisual task but rather in an improvement in visual temporal precision implies a complex relationship between multisensory and unisensory temporal processing. Rather than supporting the notion of a direct correspondence between related and temporally-dependent audiovisual tasks, the relationships observed may instead represent a dependence of visually-driven sensitivity improvements upon domain-general or unisensory timing improvements wrought by multisensory perceptual learning. The idea that these two processes may be acting in concert to produce the combination of behavioral effects observed is not novel, and in fact has been demonstrated in other models^38^ and studies^24^,^39^ of perceptual learning. Indeed, the seemingly contradictory observations that: 1) the performance improvements documented here are driven primarily by improvements in ability to correctly recognize two-flash conditions and 2) that these improvements are differentially expressed based upon the temporal structure of the auditory stimuli together warrant a complex model of interactions between visual, auditory, and multisensory temporal function. Future studies should examine the changes brought about by multisensory perceptual learning upon visual and auditory processing (see Stevenson, *et al*.^25^ as an example of visual-to-audiovisual training generalization), but should also approach the questions of generalization across low-level stimulus attributes (e.g. retinotopic location) in order to begin to identify potential loci of change relevant to each task across the information processing hierarchy^38^.

Intriguingly, the main effects described here are driven primarily by increases in participants’ abilities to discriminate between the presentation of one flash versus two flashes presented in close temporal proximity. Thus, the training effects brought about by audiovisual perceptual training largely represent changes in visual temporal performance. Other investigations into cross-modal generalization of temporally-based perceptual learning have generated mixed results. For example, transfer of learning has been shown from training on a somatosensory timing task to a corresponding auditory task if similar intervals are tested^20^, and recent work demonstrates transfer of learning from a multisensory temporal order judgment task to a visual temporal order judgment task^25^,^40^. Another study failed to demonstrate transfer of simultaneity learning either within or across different sensory modalities^41^. While the results reported here fall short of providing definitive evidence in support of the existence of a single, crossmodal or multisensory clock in its classical formulation as a pacemaker-accumulator^42^, they do join others^43^,^44^,^45^ in demonstrating the possible existence of shared components for timing perception among the sensory modalities. Indeed, these results fit well with a growing literature in support of interval-specific timing circuits that are dependent upon the time scale in question but independent of stimulus specifics, location, or modality^46^,^47^,^48^,^49^. Along these lines, future investigations should focus upon whether, as these results and cue reliability models of multisensory integration may predict^50^,^51^,^52^,^53^,^54^, the relationship between the multisensory and visual improvements described here may be causally linked.

## Methods

### 2-AFC Training

#### Subjects

Twenty-two (22) Vanderbilt undergraduate and graduate students (mean age 20.73; 11 female) took part in the 2-alternative forced-choice (2-AFC) training portion of the study. Data from this cohort of participants were obtained for a separate study (Powers *et al*.^13^) and results from training are reported in that original paper. By self-report, all participants had normal hearing and vision, and none had any history of neurological or psychiatric disorders. All procedures for all subject groups were approved by the Vanderbilt University Institutional Review Board (IRB). Additionally, all methods were carried out in accordance with the approved guidelines, and informed consent was obtained from all participants.

### 2-AFC Simultaneity Judgment Assessment

**Figure 1:**
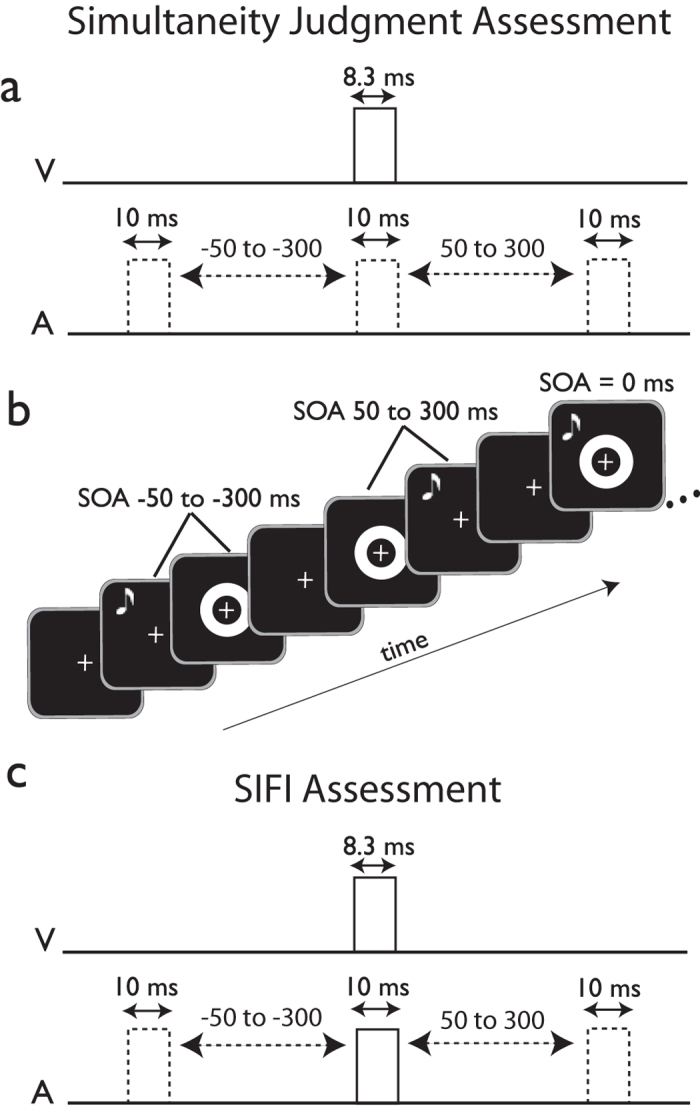
Experimental Procedures. (**a**) Temporal structure of the simultaneity judgment task. The 2-AFC version of the task consisted of one presentation of either a veridically simultaneous (SOA 0) or asynchronous (SOAs ranging from −300 ms to 300 ms by 50-ms increments) audiovisual stimulus pair, followed by a response period. The 2-IFC version presented both a simultaneous and an asynchronous pair per trial, followed by a response period. (**b**) Schematic and characteristics of stimulus presentation. (**c**) Temporal structure of the SIFI task, illusory fusion (one-flash) condition. In this condition, one flash (a solid white circle eccentrically presented below the fixation cross) is accompanied by two tones, one of which always appears simultaneously with the flash. In the two-flash condition, flashes are separated by 52 ms and the central beep is presented at the midpoint between the flashes.

**Figure 2:**
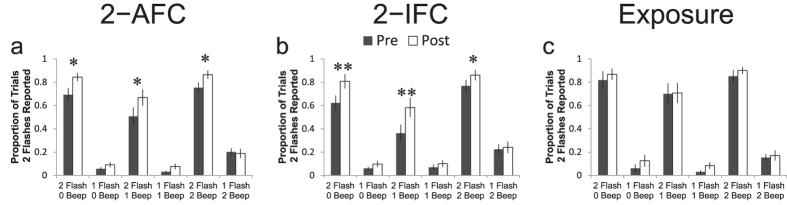
No change in reports of the SIFI illusion are seen after training, but improvements are seen in correctly reporting the number of flashes in the two-flash conditions. (**a–c**) Changes in proportion of trials in which two flashes are reported, plotted in 2-AFC (**a**), 2-IFC (**b**), and Exposure (**c**) groups as a function of training status (dark bars pre-training and light bars post-training). Increases in the probability of correctly identifying two flashes increased across all conditions, while participants continued to demonstrate relative susceptibility to both the fission (1 Flash/2 Beep) and fusion (2 Flash/1 Beep) illusory conditions relative to other 1-Flash and 2-Flash conditions, respectively.

**Figure 3:**
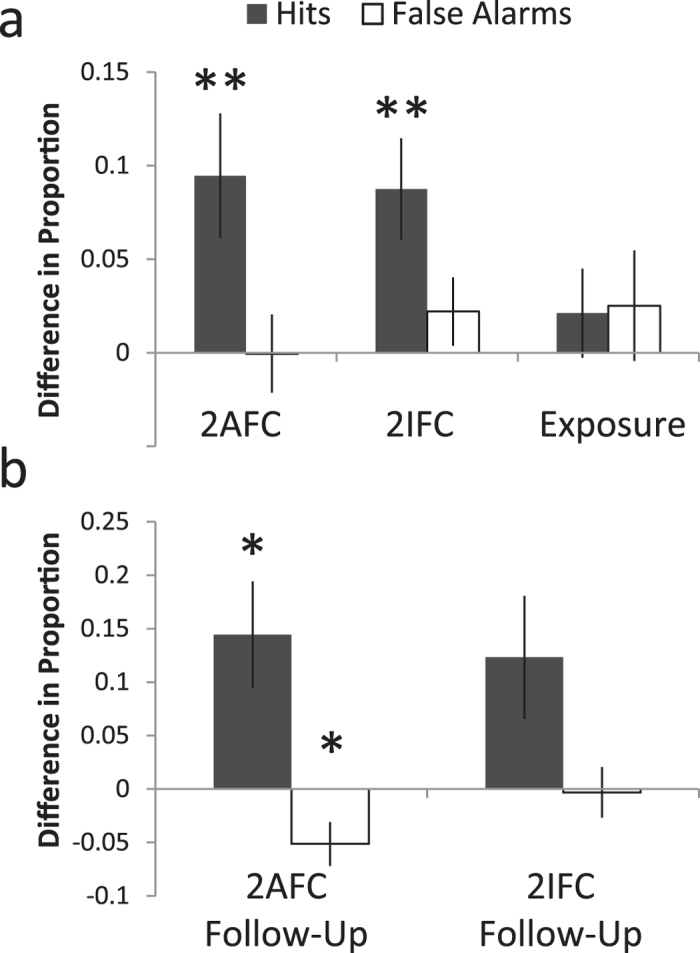
Performance improvements are driven by increased hits across all conditions. (**a**) While the 2AFC training group does exhibit a small decrease in proportion of false alarms after training, the sensitivity differences seen appear to be driven primarily by increases in hits, or correct identifications of two-flash presentations. (**b**) As seen in the assessment immediately following training, the shift on 1-week follow-up assessment appears to be primarily driven by an increase in hits, although smaller decreases in false alarms are also present. Error bars indicate one SEM; *p < 0.05; **p < 0.01.

**Figure 4:**
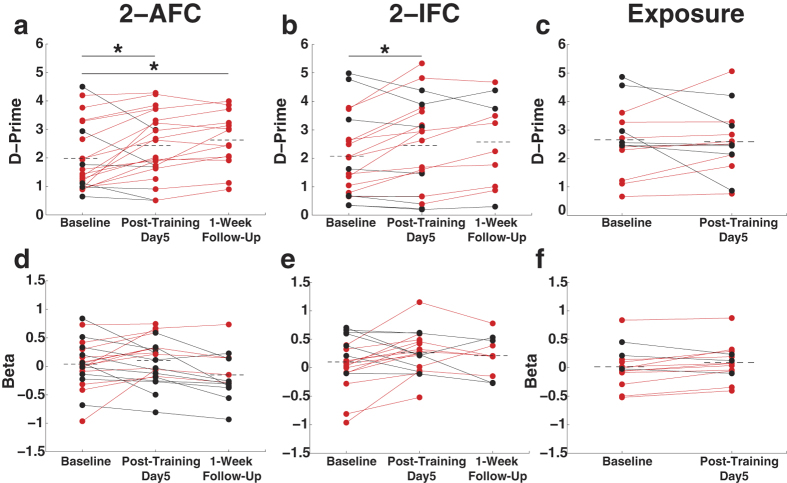
Multisensory perceptual training results in changes in sensitivity on the SIFI task, but no changes in response bias. Changes in d-prime (**a–c**) and beta (**d–f**) values in the 2-AFC (**a**), 2-IFC (**b**), and Exposure (**c**) groups. Plots represent individual subject measures at baseline, post-training/exposure, and 1-week follow-up assessments. Red dots indicate individuals whose measures demonstrate increases from baseline. Black dots indicate individuals whose measures demonstrate decreases from baseline. Dotted lines connote group means at each assessment. While both 2-AFC and 2-IFC training groups demonstrated significant increases from baseline to post-training assessments, Exposure participants did not. 2-AFC participants also demonstrated a significant increase from baseline on 1-week follow-up. In contrast to the d-prime measures, no differences in beta measures were detected in any of the three groups. ^*^p < 0.05.

**Figure 5:**
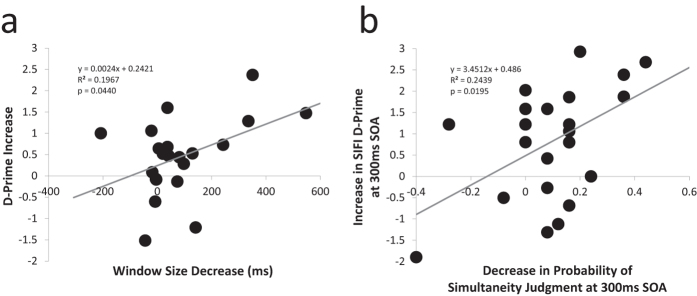
Increases in d-prime on the SIFI task correlate with measures of improvement in audiovisual simultaneity judgment. (**a**) Relationship between magnitude of window narrowing exhibited in the simultaneity judgment task and change in sensitivity from pre- to post-training in 2-AFC training participants. (**b**) Correlation between decreased probability of simultaneity judgment at the 300ms SOA and increased d-prime at the 300 SOA on the separate SIFI task.

In this task (Fig. 1a,b), participants judged whether the presentation of an auditory stimulus and a visual stimulus was ‘simultaneous’ or ‘non-simultaneous’ by pressing 1 or 2, respectively, on a response box. For details of stimulus characteristics and task structure, please see^13^.

### Sound-Induced Flash Illusion (SIFI) Task

Participants completed the SIFI task^28^,^29^,^55^ once directly after the baseline simultaneity judgment assessment and once again after the completion of the final simultaneity judgment assessment on Day 5. In this task, participants are presented with one or two flashes paired with zero, one, or two beeps. Flashes were 8.5 ms in duration, and consisted of a white circle with an area of 12.6 cm^2^ presented on a black background one centimeter below the fixation cross. Beeps consisted of a temporally-ramped 5000 Hz pure tone of 8 ms duration. In the two flash/one beep condition, the flashes were separated by 50 ms and the beep was presented at the midpoint between the two flashes. Similarly, in the two flash/two beep condition, the timing between flashes remained constant and one beep was always presented at the midpoint between the two flashes, with the other preceding or following it by an SOA ranging from 50 to 300 ms. In the illusion-inducing one flash/two beeps condition, one beep always occurred simultaneously with the flash onset, while one either preceded or followed that onset by SOAs ranging from 50 to 300 ms. This condition typically induces the perception of two flashes although only one appears, and the strength of this illusion varies with SOA^29^. Each of these conditions occurred an equal number of times so as not to introduce a response bias. After each trial, participants responded by button-press to indicate the number of flashes they had perceived. In the SIFI assessment task there were 300 total trials (10 cycles × 30 trials/cycle) with an equal distribution of each condition.

### 2-AFC Simultaneity Judgment Training

The training task differed from simultaneity judgment assessments in that after making a response, the subject was presented with either the phrase “Correct!” paired with a happy face, or “Incorrect” paired with a sad face corresponding to the correctness of their choice. These faces (happy = yellow, sad = blue, area = 37.4 cm^2^) were presented for 500 ms in the center of the screen. The white ring and fixation were of the same size and duration as in assessment trials. Only SOAs between −150 and 150 ms, broken into 50 ms intervals, were used during the training phase. Additionally, in this phase the SOAs were unequally distributed: the veridical simultaneous condition had a 6:1 ratio to any of the other 6 non-simultaneous conditions. In this way there was an equal likelihood of simultaneous/non-simultaneous conditions, minimizing concerns about introducing a response bias. The training phase consisted of 120 trials (20 cycles × 6 trials/cycle). See Fig. 1a,b for illustrations of the temporal relationship between stimuli.

### 2-AFC Training Protocol

Training occurred over 5 hours (1 hour per day) during which participants took part first in a pre-training simultaneity judgment assessment, next in one SIFI assessment, then in 3 shorter simultaneity judgment training runs, followed by a post-training simultaneity judgment assessment. An additional baseline assessment was performed at the start of the study for each subject, followed by the typical training day; this was designed to detect any practice effects that may have resulted from completion of the assessment itself.

### Follow-Up Assessment

After one week without training, a subset of the training cohort described above (n = 14, 6 female; mean age = 21.14) returned to the lab and underwent one simultaneity judgment assessment and one SIFI assessment without any training.

### 2-AFC Exposure

#### Subjects

Fourteen (14) Vanderbilt undergraduate and graduate students (mean age 19.5; 4 female) underwent the 2-alternative forced-choice (2-AFC) exposure portion of the study. As with the 2-AFC training group data, data from this cohort of participants represents data obtained for a separate study (Powers *et al*.^13^). All participants had self-reported normal sight and hearing, and none had any personal or family history of neurological or psychiatric disorders.

#### Exposure Protocol

The exposure portion of the study differed from the 2-AFC training protocol only in that instead of the training blocks, participants underwent 2-AFC exposure blocks of the same length. Thus, all participants in both cohorts took part in the same number of 2-AFC simultaneity judgment and SIFI assessments. The details of the exposure sessions are outlined below.

### 2-AFC Exposure

In the interest of maintaining attention, the 2-AFC exposure blocks were designed as an oddball task wherein participants were exposed to the same audiovisual pairs used in the simultaneity judgment training sessions but were instructed to press a button when they saw a red ring. As in the simultaneity judgment training sessions, the veridical simultaneous condition had a 6:1 ratio to any of the other 6 non-simultaneous conditions. Oddballs occurred with the same probability across all conditions, and were 1/10 as likely to appear as the standard. The rings and fixation were of the same dimensions and duration as in the assessment trial; the tone was identical to that presented during the simultaneity judgment assessment and training sessions. A range of SOAs between −150 and 150 ms, in steps of 50-ms intervals, were used for this task.

### 2-IFC Training

#### Subjects

Twenty (20) Vanderbilt undergraduate and graduate students (mean age 20.3; 14 female) underwent the 2-interval forced-choice (2-IFC) training portion of the study. As with data from the other cohorts, data from this cohort of participants represents a subset of that obtained for a separate study (Powers *et al*.^13^). All participants had normal hearing and vision by self-report, and none had any personal or close family history of neurological or psychiatric disorders.

### 2-IFC Simultaneity Judgment Assessment

The 2-IFC simultaneity judgment assessment employed precisely the same stimuli as those used in the 2-AFC task. In this task, however, participants were presented with two audiovisual pairs, one with an SOA of zero (simultaneously-presented) and one with a non-zero SOA (non-simultaneously presented). Presentations were separated by 1 second, during which a fixation cross alone was presented. Participants were asked to indicate as quickly as possible by button-press which interval (first or second presentation) contained the flash and beep that happened at the same time. Simultaneous pairings were as likely to be presented in the first interval as in the second, and a simultaneous-simultaneous catch trial condition was present in equal representation to other SOAs.

### 2-IFC Simultaneity Judgment Training

The training phase of the 2-IFC portion of the study was identical to that of the assessment phase with two exceptions: 1) in the same manner described in the 2-AFC training, participants were given feedback as to the accuracy of their responses after each trial; 2) as in the 2-AFC simultaneity judgment training protocol, the range of SOAs presented during training (−150 ms to 150 ms by 50-ms increments) was restricted in training as compared to assessment (−300 ms to 300 ms). However, unlike the 2-AFC version of this training, the ratio of simultaneous to non-simultaneous presentation was always 1:1.

### 2-IFC Training Protocol

Participants underwent training in five 1-hour blocks (one hour per day) on the 2-IFC version of the simultaneity judgment task. Each day’s 2-IFC training began with a simultaneity judgment assessment followed by three shorter blocks of training, and ended with a post-training simultaneity judgment assessment.

### Follow-Up Assessment

A subset of the 2-IFC training cohort described above (n = 10, 7 female; mean age = 20.9) returned to the lab one week after cessation of training and underwent one simultaneity judgment assessment and one SIFI assessment without any training.

### Data Analysis

All data were imported from E-Prime 2.0 text files into MatLab 7.7.0.471 R2008b (The Mathworks, Inc., Natick, MA) via a custom-made script for this purpose. Individual subject raw data were used to calculate the mean probability of simultaneity judgment (2-AFC), accuracy (2-IFC), and proportion of trials at which two flashes were reported (SIFI) at each SOA for all assessments. These means were then analyzed in multiple ways as summarized in the Results section.

### Estimation of Window Size

Mean data from each individual were fit with two sigmoid curves generated using the MatLab *glmfit* function, splitting the data into left (auditory presented first) and right (visual presented first) sides and fitting them separately. For the 2-AFC tasks, the criterion at which to measure the breadth of the temporal window was equal to 75% of the maximum data point at baseline assessment. For the 2-IFC task, this criterion was set at half the distance between individuals’ lowest accuracy point at baseline assessment and 1 (also ~75% accuracy). These criteria were then used to assess the breadth of the distributions produced by each individual’s assessment data throughout the duration of the training period. Distribution breadth was then assessed for both the left side (from zero to the left-most point at which the sigmoid curve crossed the criterion line) and the right side (from zero to right intersection point) and then combined to get an estimation of total distribution width. This measure was then used as a proxy for the size of each individual’s window at each assessment.

### Signal Detection Analysis

In order to determine whether any changes in SIFI performance were the result of a true increase in perceptual sensitivity (d′) or a shift in response bias (β), a signal detection analysis was performed. Perceptual sensitivity (d′) was defined as the ability to discriminate between one flash and multiple flashes^35^,^55^. These parameters were calculated per individual in the following manner:


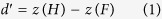



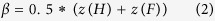


where *z*(p) indicates the inverse of the cumulative normal distribution corresponding to the response proportion *p*. H (hit) denotes correct detection of multiple flashes, and F (false alarm) indicates an incorrect report of multiple flashes.

## Additional Information

**How to cite this article**: Powers, A. R. III *et al*. Generalization of multisensory perceptual learning. *Sci. Rep.* **6**, 23374; doi: 10.1038/srep23374 (2016).
